# Supplementary material for: Expression and splicing mediate distinct biological signals
Source: BMC Biol. 2023 Oct 20;21:220. doi: 10.1186/s12915-023-01724-w (PMC10588054; doi:10.1186/s12915-023-01724-w)
Supplement: Supplementary file 1 — Additional file 1: Figure S1. Summary of the manual literature review of articles from 2020 that analyzes RNA-seq data (see Methods). Figure S2. Simulation of how the enrichment score calculated based on gene-set enrichment analysis of both splicing and expression differs (x-axis) depending on which group of genes is enriched (y-axis). Boxplot summarizes 1000 simulations per group. Figure S3. Histogram of the Spearman’s correlations between gene-set enrichment scores for gene sets significantly (FDR-adjusted P-value of < 0.05) enriched among differentially expressed or differentially spliced genes. Correlations were computed separately for gene sets enriched among either differentially spliced or expressed genes or both (sub-plots). Figure S4. For each comparison and data subset (y-axis), the median differences between relative risks of gene sets enriched among differentially expressed and spliced genes as the percent change of the smallest risk. Data subsets are gene sets that were significantly (FDR-adjusted P-value of < 0.05) enriched among differentially spliced genes, expressed genes, or both. Figure S5. Same as main Fig. 1B-C but created from the limma-based analysis. A) The distribution of false positives, i.e., the number of significantly differentially expressed genes only found when not corrected for confounders, across the 199 comparisons. B) Histogram of the false discovery rate when not correcting for confounders. Significance is defined as having an FDR-adjusted p-value of <0.05. Figure S6. Same as main Fig. 2 but created from the limma-based analysis. Differential splicing is just as frequent as differential expression. A) The number of significant genes for each comparison across analyses. B) For each analysis, the fraction of genes tested that were deemed significant. C) The fraction of differentially expressed genes that were also differentially spliced. D) Within the genes that are both differentially expressed and spliced, we calculated the fract [file 12915_2023_1724_MOESM1_ESM.pdf]

Expression and Splicing Mediate Distinct Biological Signals

Søren Helweg Dam[1], Lars Rønn Olsen[1], Kristoffer Vitting-Seerup[1,*]

Additional File 1: Supplementary Figures

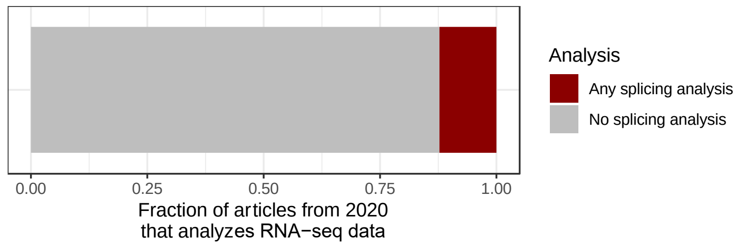

Figure S1: Summary of the manual literature review of articles from 2020 that analyzes RNA-seq data (see Methods).

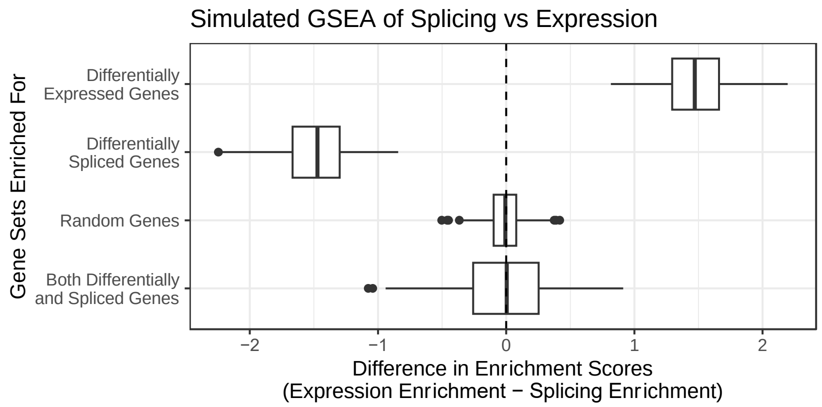

Figure S2: Simulation of how the enrichment score calculated based on gene-set enrichment analysis of both splicing and expression differs (x-axis) depending on which group of genes is enriched (y-axis). Boxplot summarizes 1000 simulations per group.

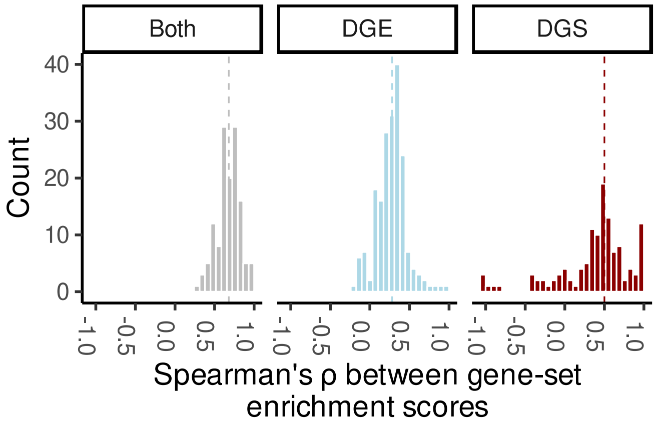

Figure S3: Histogram of the Spearman's correlations between gene-set enrichment scores for gene sets significantly (FDR-adjusted P-value of <0.05) enriched among differentially expressed or differentially spliced genes. Correlations were computed separately for gene sets enriched among either differentially spliced or expressed genes or both (sub-plots).

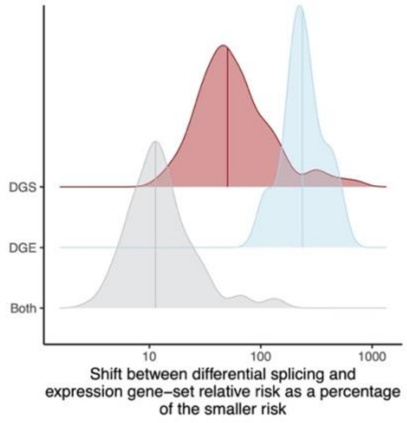

Figure S4: For each comparison and data subset (y-axis), For each comparison, the median differences between relative risks of gene sets enriched among differentially expressed and spliced genes as the percent change of the smallest risk. Data subsets are gene sets that were significantly (FDR-adjusted P-value of <0.05) enriched among differentially spliced genes, expressed genes, or both.

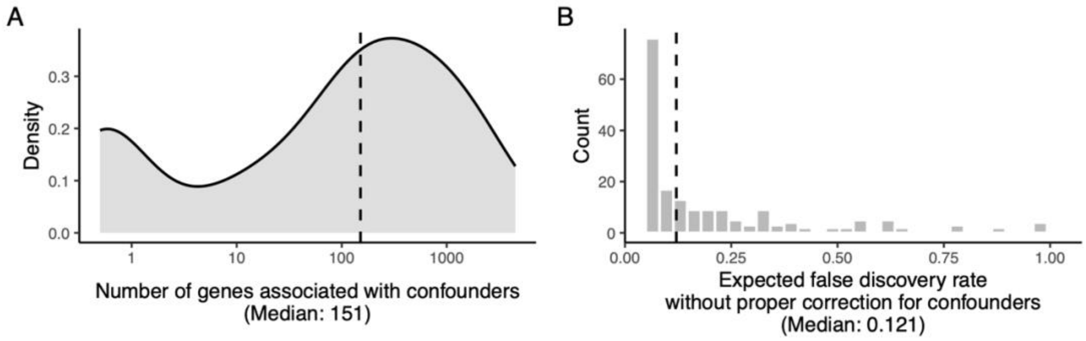

Figure S5: Same as main figure 1B-C but created from the limma based analysis. **A**) The distribution of false positives, i.e., the number of significantly differentially expressed genes only found when not corrected for confounders, across the 199 comparisons. **B**) Histogram of the false discovery rate when not correcting for confounders. Significance is defined as having an FDR-adjusted p-value of <0.05.

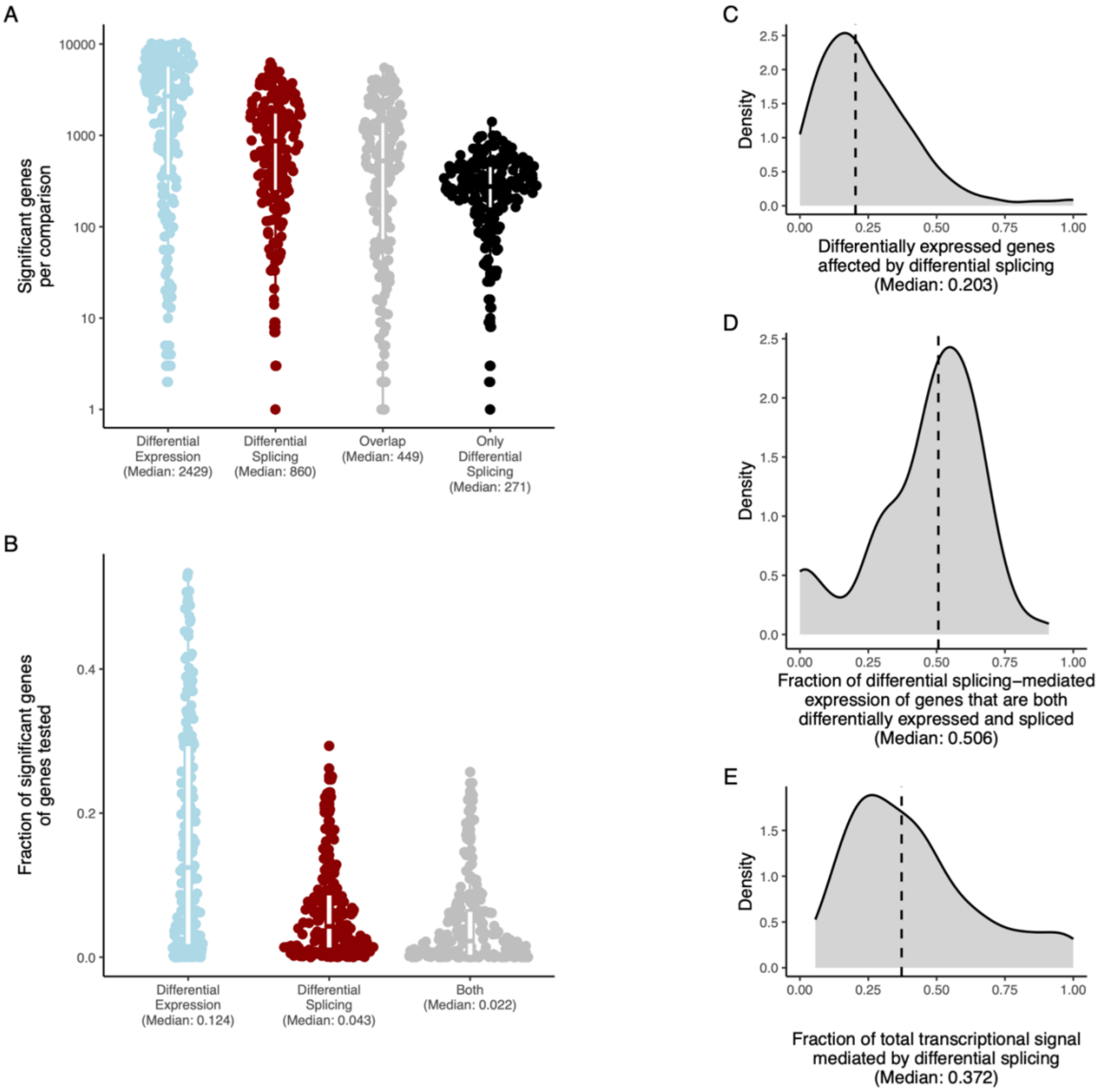

Figure S6: Same as main figure 2 but created from the limma based analysis. Differential Splicing is just as Frequent as Differential Expression. **A**) The number of significant genes for each comparison across analyses. **B**) For each analysis, the fraction of genes tested that were deemed significant. **C**) The fraction of differentially expressed genes that were also differentially spliced. **D)** Within the genes that are both differentially expressed and spliced, we calculated the fraction of the gene expression that is contributed by differentially spliced transcripts. For each analysis, we extracted the median. **E**) The number of differentially spliced genes as a fraction of the total number of genes either differentially spliced or expressed genes (total transcriptional signal). Across all panes, significance is defined as having an FDR-adjusted P-value of <0.05. Medians are indicated for all plots.

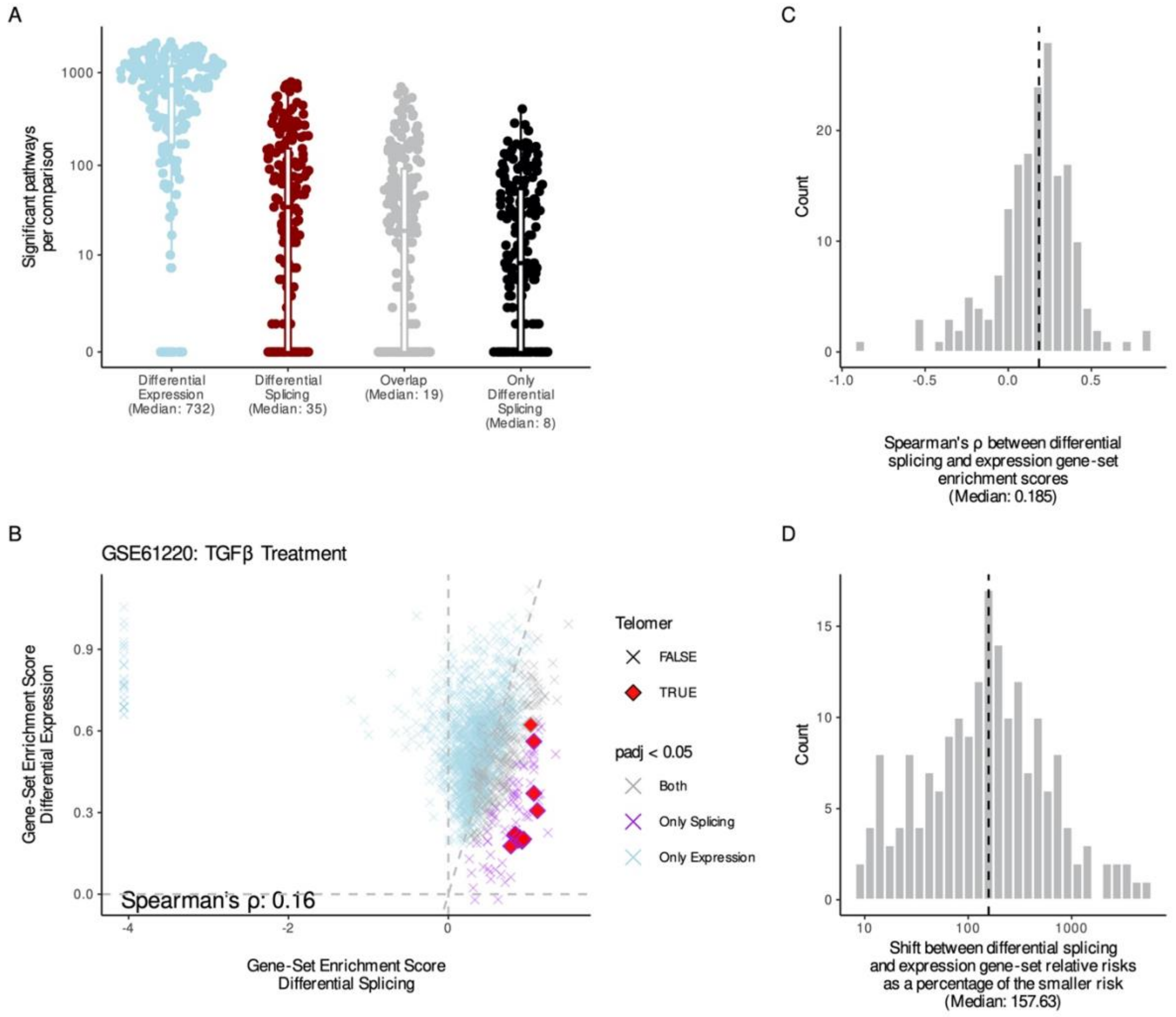

Figure S7: Same as main figure 3 but created from the limma based analysis. Splicing and expression regulate distinct biological processes. **A**) The number of gene sets significantly enriched among genes from either analysis across comparisons. **B**) Results from the Tian *et al*. [26] study showing the gene-set enrichment scores of gene sets enriched among the differentially spliced (x-axis) and differentially expressed (y-axis) genes. Only gene sets significantly enriched among differentially spliced or differentially expressed genes (indicated by color) are shown. The shape highlights gene sets where the name contains the word "telomer". Spearman's correlation is indicated in the lower left corner. **C**) Histogram of the Spearman's correlations between gene-set enrichment scores for gene sets significantly enriched among differentially expressed or spliced genes. **D**) For each comparison, we calculated the median differences between the relative risks of gene sets enriched among differentially expressed and spliced genes as the percent change of the smallest risk score
